# Maternal high fat and high sugar diet impacts on key DNA methylation enzymes in offspring brain in a sex-specific manner

**DOI:** 10.1111/jne.70046

**Published:** 2025-05-15

**Authors:** Kahyee Hor, Laura Dearden, Emily Herzstein, Susan Ozanne, Giles Hardingham, Amanda J. Drake

**Affiliations:** 1 Centre for Reproductive Health, Institute for Regeneration and Repair, University of Edinburgh, Edinburgh, UK; 2 University of Cambridge Metabolic Research Laboratories, Institute of Metabolic Science, Addenbrooke's Hospital, Cambridge, UK; 3 UK Dementia Research Institute, University of Edinburgh, Edinburgh Medical School, Edinburgh, UK

**Keywords:** DNA methylation, epigenetics, neurodevelopment, obesity, pregnancy

## Abstract

Maternal obesity associates with an increased risk of offspring neurodevelopmental disorders. Although the underlying mechanism(s) remain unclear, evidence suggests a role for altered DNA methylation. We utilized a murine model of diet-induced obesity to investigate the impact of maternal obesity on the offspring brain transcriptome and DNA methylation. C57Bl/6 dams were fed high-fat high-sugar (HFD, *n* = 7) or control (CON, *n* = 7) diets. Maternal obesity/hyperglycemia associated with offspring growth restriction, with brain-sparing specifically in females. Postnatal hypoglycemia was seen in HFD males, but not females. The 3′ RNA-sequencing revealed perturbations in metabolic and cell differentiation pathways in neonatal male and female off-spring frontal cortex and cerebellum. Compared with controls, HFD males, but not females, had lower cortical and cerebellar DNMT gene and protein expression, and reduced cerebellar TET enzyme mRNA. Whilst female offspring had lower cerebellar 5-methylcytosine (5mC) and 5-hydroxymethylcytosine (5hmC) than males, there were no effects of HFD on 5mC/5hmC in cortex or cerebellum in either sex. Our data suggest that maternal obesity has sex-specific effects on fetal neurodevelopment, including enzymes involved in DNA methylation/demethylation. These mechanisms may play a role in the increased risk of neurodevelopmental disorders following obese/diabetic pregnancies, including increased male susceptibility to these disorders.

## 1 Introduction

The prevalence of obesity is increasing across the world with important consequences for global disease burden and economic output.^1,2^ Worldwide, the proportion of women with a body mass index (BMI) of 25 kg/m^2^ or greater is estimated to be around 38.0%.^3^ This includes pregnant women, such that ~1 in 5 pregnant women may be obese.^4^ Some countries report higher prevalence data including the United States where rates of pre-pregnancy overweight (BMI 25.0–29.9 kg/m^2^) and obesity (BMI ≥30 kg/m^2^) have been estimated at 25.8% and 25.6%, respectively.^5^ Data from the Euro-Peristat Project (2015) reported that 30%–50% of pregnant women in Europe were overweight and 8%–26% obese.^6^ The increasing prevalence of obesity in adolescents and young adults^1,7^ emphasizes the need for global public health measures to tackle obesity if there is not to be a continued rise in obesity prevalence amongst pregnant women. Indeed, a recent study has predicted that a lack of effective interventions may result in an increase of overweight and obese individuals by 44% and 45%, respectively, by 2030.^1^

Whilst maternal obesity during pregnancy is associated with significant immediate risks to the mother and offspring^8^ it also has implications for both maternal and offspring long-term health.^9^ During the antenatal and early postnatal period, exposure to “adverse” environments can impact on key developmental processes in the offspring, leading to long-lasting effects into adulthood.^10–13^ The associations between maternal obesity and the long-term risk of non-communicable diseases in her offspring, including poor cardiometabolic health, cancer, and respiratory diseases, are widely recognized.^14–16^ There is also growing evidence for an association between higher maternal BMI and diabetes in pregnancy and an increased risk of neurodevelopmental disorders in her offspring.^17^ Evidence from epidemiological studies suggests that maternal obesity, gestational and/or type 2 diabetes, and increased gestational weight gain are associated with an increased risk of autism spectrum disorders (ASD), attention deficit-hyperactivity disorder (ADHD) and impaired cognitive function in her children.^18–21^ A meta-analysis by Wang et al.^19^ identified a 16% increase in offspring risk of ASD for every 5 kg/m^2^ increase in maternal BMI. Gestational diabetes diagnosed before 26 weeks' gestation and type 2 diabetes associates with a relative risk of 1.42 (95% CI: 1.16–1.75) and 1.33 (95% CI: 1.07–1.66), respectively, of offspring developing ASD.^20^ These data are supported by studies in animal models which confirm that maternal diet-induced obesity can affect offspring neurodevelopment.^22^

Human brain development begins in the third gestational week and continues throughout gestation, involving a complex network of molecular mechanisms and changes in gene expression.^23^ Kang et al. published a detailed analysis of a gender-specific genome-wide exonlevel transcriptomic data from 16 human brain regions ranging from the embryonic period to late adulthood.^24^ Genes involved in key neurodevelopmental processes, including cell proliferation, dendrite development, synaptogenesis, and myelination, were interrogated, and transcriptional trajectories identified a highly dynamic process in these processes during the fetal period. Cell proliferation was at its peak in the neocortex and cerebellum during fetal development, and this progressively reduced over time. However, synaptogenesis and myelination exponentially increased in utero and peaked at the time of birth, and they remained at their maximum transcriptional velocity into adulthood. Exploration of the transcription of glycolytic genes identified an increase in glycolytic gene transcription in the cortex over the course of fetal development, whereas transcriptional activity peaked in utero in the cerebellum,^25^ suggesting that the fetal frontal cortex and cerebellum are potentially vulnerable to in utero exposure to hyperglycemia during obese and/or diabetic pregnancies. Indeed, aberrant structural organization and cellular connectivity in both the frontal cortex and cerebellum have been implicated in childhood-onset neurodevelopmental disorders, including autism spectrum disorders and attention deficit-hyperactivity disorders.^26–30^

There is increasing evidence that dynamic changes in DNA methylation (5-methylcytosine, [5mC] and 5-hydroxymethylcytosine [5hmC]) are crucial for normal in utero brain development.^31,32^ For example, DNA methylation associates with chromatin accessibility, particularly at regulatory sites, suggesting a key role in the determination of cell identity and function.^33^ Dynamic changes in 5mC/5hmC levels also occur during neurogenesis^31^; for example, neural progenitor cells and immature neurons have lower levels of 5hmC in comparison to differentiated cortical plate neurons.^31^ These dynamic changes in DNA methylation/hydroxymethylation, which are important for normal neurodevelopment, are facilitated by the DNA methyltransferase (DNMT) and Ten-eleven translocation (Tet) enzymes.^34–37^

Epigenome-wide association studies of peripheral blood from off-spring of women with gestational diabetes and obesity during pregnancy have identified differential methylation of genes involved in lipid metabolism and endocrine functioning, which may be involved in the increased susceptibility to obesity and impaired glucose tolerance.^38^ However, the extent to which the maternal metabolic milieu influences the epigenome of the developing fetal brain and the extent to which this contributes to psychiatric and neurodevelopmental disorders is less well understood.^39,40^ In this study, we used a mouse model of maternal obesity to study the effects on the developing brain transcriptome and DNA methylome in male and female offspring.

## 2 Methods

### 2.1 Animals

All animal procedures were performed in accordance with the Animals (Scientific Procedures) Act 1986. Experimental protocol and methods (Experiment ID: 84-LF2-18 and 644-LF2-18) were approved by the United Kingdom Home Office and local ethics committee. Animals were maintained in a controlled environment with set humidity (55%), temperature (21°C–22°C) and lighting (12-h light/12-h dark).

Female C57Bl/6 mice (in house stock, Harlan, UK) aged 3 weeks to 3 weeks + 3 days were randomly allocated to either “high-fat diet” (HFD: 45% fat; Special Diets Services [SDS, 824053]), supplemented with “high sugar” condensed milk (Carnation Sweetened Condensed Milk [31% sugars, 6% fat] with added vitamin premix [SDS 829912], 62.5 mg per 30 mL) or “control diet” (CON: Special Diets Services [RM1(P) 801151]) as previously described.^41^ Each cage housed two females with ad lib access to food and water during the experimental period.

**Figure 1:**
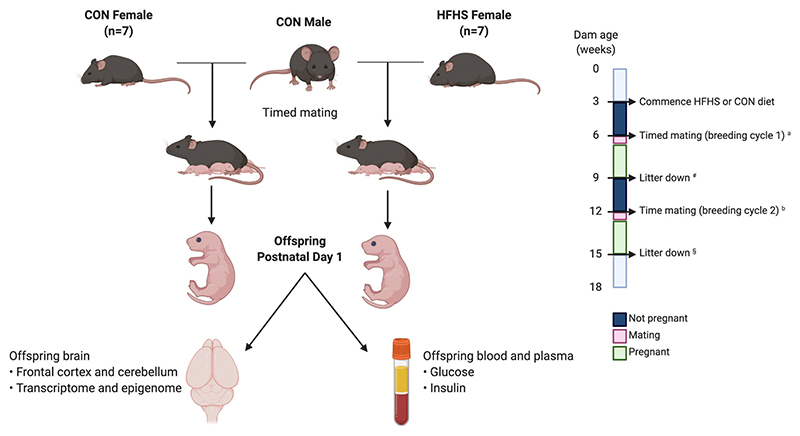
Mouse model of high-fat high-sugar diet (HFD)-induced obesity. *N* = 7 in each diet group, and offspring from the second mating cycle were retrieved for dissection and further analysis. ^a^Breeding cycle 1 confirmed dam breeding ability. ^b^Only dams that successfully fell pregnant in breeding cycle 1 were included in breeding cycle 2 (proven breeders). ^#^Offspring from breeding cycle 1 was killed on postnatal day 1. ^§^Offspring from breeding cycle 2 were retrieved on postnatal day 1 for downstream experiments (Created using BioRender).

A first mating cycle was performed following 3 weeks of being on the diet, irrespective of weight, to confirm breeding ability (CON *n* = 17, HFD *n* = 16). Each mating cage consisted of 2 dams and 1 stud male, which had previously been maintained on standard chow (Special Diets Services [RM1(E) 801003]). Cages were checked daily for the presence of vaginal plugs. Mice that had successful pregnancies within 12 days (i.e., proven breeders) were included in the ongoing experiment (*n* = CON 17; HFD 16). Dams in the HFD group, which reached a weight >30 g, were then included in a second mating cycle to generate pups used for the experiment (HFD dams with successful first mating cycle and >30 g before second mating cycle, *n* = 10. HFD dams with successful second mating cycle, *n* = 7). During this second mating cycle, dams were weighed on embryonic days (E)0.5, E5, E10, and E18, and on postnatal days (PN)1 and 10 (Ohaus Scout STX2201 Portable Balance). Tail nick blood was collected from dams on E0.5, E10, and E18, PN1 (after pups were removed) and PN10. All blood tests were performed between 0930 and 1100 h, and animals had usual access to diet and water before, during, and after blood sampling (see Figure 1).

Offspring born following the second mating cycle were retrieved on PN1 and killed by decapitation. Pup weight was measured and sex was determined by checking anogenital distance. The brain was removed, weighed, and then micro-dissected using a dissecting microscope (Stemi 2000, Zeiss) to obtain the frontal cortex and cerebellum. All tissues were snap frozen and stored at −80°C until ready for use.

Blood from dams (tail nick) and pups (trunk blood) was analyzed for blood glucose concentrations using the Accu-Chek Performa Nano Blood Glucose System (Roche, UK). Plasma was obtained following centrifugation (12,000*g* for 10 min at 4°C) and stored at −80°C. Off-spring and dam plasma insulin concentrations were quantified using the Ultrasensitive Mouse Insulin ELISA Kit (Mercodia).

### 2.2 Transcriptomic analysis

RNA was extracted from the frontal cortex and cerebellum from one male and one female offspring randomly selected from each litter (i.e., male offspring, *n* = 7/diet group; female offspring, *n* = 7/diet group) using QIAzol Lysis Reagent and the QIAGEN RNeasy Kit (QIAGEN, UK), and stored at −80°C. RNA quality was assessed with gel electrophoresis (18S and 21S bands) and quantity assessed using a spectrophotometer (Nanodrop 2000, Thermo Fisher Scientific). For quantitative qPCR, cDNA was generated following reverse transcription of RNA using the High Capacity cDNA Reverse Transcriptase Kit (QIAGEN, UK). qPCR was performed using either SYBR Green or probe-based assay on a LightCycler 480 Instrument (Roche, West Sussex, UK), followed by melting curve analysis. Primers were designed using the Roche Universal ProbeLibrary Assay Design Center (Table 1).

For RNA-sequencing, library preparation and 3′ RNA-sequencing were performed at the Genetics Core, Edinburgh Clinical Research Facility, University of Edinburgh (*n* = 7 for each sex and diet group). Single read (1× 75 bp) sequencing was performed using the NextSeq 500/550 High-Output v2.5 (75 cycle) Kit (Illumina) on the NextSeq 550 platform (Illumina). The libraries were combined in 2 equimolar pools of 42 libraries and run across 2× NextSeq 500/550 High Output v2.5 Flow Cells (Illumina). Raw data were transferred in “.bcl” format from the NextSeq 550 instrument to a computer running Linux and used to generate demultiplexed FASTQ files using the Bcl2fastq2 v2.17.1.14 software (Illumina). The lane-splitting feature on this software was disabled to create a single FASTQ file for each library. QuantSeq FWD data analysis was performed on the Bluebee Genomics Platform (1.10.16, www.bluebee.com). The generated raw reads were trimmed and aligned to the Encyclopedia of DNA Elements (ENCODE) mouse reference genome (mm9) using the Spliced Transcript Alignment to a Reference (STAR) aligner. Raw gene counts for each individual sample were produced following in-built quality control steps. Analysis was performed on R (version 3.6.2, https://www.r-project.org/), with its associated packages (https://cran.r-project.org/). The edgeR platform (version 3.28.1) was used to normalize read counts and determine differentially expressed genes (https://bioconductor.org/packages/release/bioc/html/edgeR.html). A full list of R packages has been included in Supporting Information S1. Functional enrichment analysis of differentially expressed genes was performed using Gene Set Enrichment Analysis (GSEA) Software (v4.1.0).^42^

### 2.3 DNA extraction and ultra-performance liquid chromatography (UPLC) for 5mC/5hmC

DNA was extracted from frozen dissected offspring brain sections using the QIAGEN DNeasy Blood and Tissue Kit (DNA was obtained from male cortex, CON *n* = 7, HFD *n* = 5.; male cerebellum, CON *n* = 7, HFD *n* = 5. Female cortex, CON *n* = 7, HFD *n* = 6; female cerebellum, CON *n* = 4, HFD *n* = 4). DNA quality and quantity were assessed with a spectrophotometer (Nanodrop 2000, Thermo Fisher Scientific). The samples were processed at the Mass Spectrometry Facility, Institute for Genetic and Molecular Medicine (IGMM), University of Edinburgh as previously described.^43^

### 2.4 Western blot

Protein was extracted from defrosted brain tissue sections (*n* = 3 for each sex and diet group) using lysis buffer (RIPA buffer) with a protease inhibitor complex. Protein concentration was quantified using a modified Lowry assay (DC Protein Assay Kit, Bio-Rad Laboratories) according to the manufacturer's protocol. Extracted protein (30 μg/sample) was resolved by SDS-PAGE, using Novex Wedgewell (4%–12%) pre-loaded gels (Thermo Fisher). Protein bands were then transferred onto polyvinylidene difluoride (PVDF) membranes using the Trans-Blot Turbo Blotting System (Bio-Rad). Membranes were blocked in 5% skimmed milk (in Tris-buffered saline buffer with 0.2% Tween, TBS-T) and incubated overnight in primary antibodies (Anti-DNMT1 antibody, Abcam ab188453, 1:1000. Anti-DNMT3A antibody, Abcam ab188470, 1:2000. Anti-beta Actin antibody, Proteintech 66,009, 1:10000. All antibodies were diluted in 3% BSA in Tris-buffered saline) at 4°C. Membranes were then incubated in secondary antibodies (IRDye 800CW and IRDye 680CW (LI-COR, anti-mouse and anti-rabbit IgGs), diluted at 1:10,000 dilution in 3% BSA in TBS with and 0.01% SDS) for 1 h at room temperature. Membranes were scanned using the Li-cor Odyssey Clx Imaging system, and protein bands were analyzed using Image Studio Light software (LI-COR, version 5.2.5). Protein bands of interest were normalized against beta-Actin.

### 2.5 Statistical analysis

Data from 3′-RNA sequencing were deemed statistically significant if they met the following criteria: (1) false discovery rate (FDR) <0.05, and (2) adjusted *p*-value <.05. These values were calculated using the edgeR package on R (version 3.6.2). Statistical analysis was performed using GraphPad Prism software (version 9.0.0 for macOS, GraphPad Software LLC) and statistical tests have been described within the respective result sections. Where appropriate, *Q*–*Q* plots were used to test the assumption of normality; Tukey's multiple comparison test was performed, and analyses were considered statistically significant when *p*-value was <.05. Unless otherwise stated, all values are presented as mean ± SEM.

## 3 Results

### 3.1 Dams on high-fat diet were obese and hyperglycemic but had lighter pups

**Figure 2:**
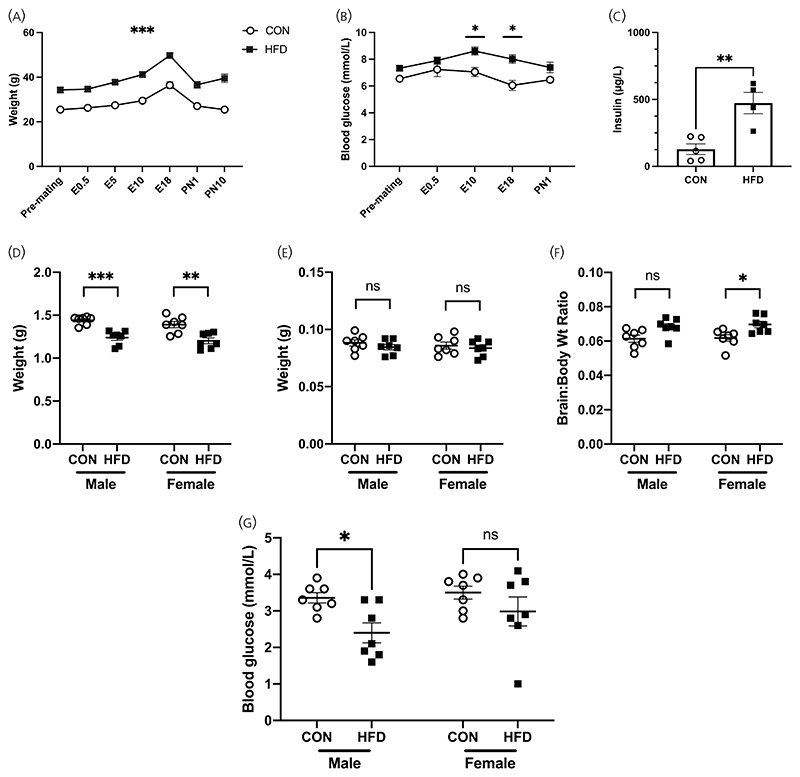
Effects of HFD on dams and offspring. (A–C) Dam characteristics. (A) Repeated measures ANOVA showed that HFD dams (black squares) were consistently heavier than CON dams (blank circles) during the pre-mating, gestational and postnatal period (*F*(6,69) = 4.754, *p* = .0004). (B) HFD dams were more hyperglycemic than CON (repeated measures ANOVA, *F*(4,57) = 1.505, *p* = .213). (C) HFD dams had higher plasma insulin than CON (unpaired *t*-test, *p* = .004). (D–G) Offspring characteristics at postnatal day 1. (D) In both male and female offspring, HFD offspring were lighter than CON (Male CON 1.44 ± 0.02 g, HFD 1.24 ± 0.03 g, *p* = .0006; Female CON 1.39 ± 0.04 g, HFD 1.20 ± 0.03 g, *p* = 012. *F*(1,24) = 41.03, *p* < .0001). (E) There were no differences in brain weight between HFD versus CON offspring (Male CON 0.088 ± 0.003 g, HFD 0.085 ± 0.002 g, *p* = .767; Female CON 0.086 ± 0.003 g, HFD 0.084 ± 0.002 g, *p* = .943. *F*(1,24) = 0.3688, *p* = .549). (F) There were no differences in brain:body weight ratio between HFD versus CON male offspring (CON 0.061 ± 0.002 g, HFD 0.069 ± 0.002 g, 0.074). However, HFD female offspring had a higher brain:body weight ratio than CON (CON 0.062 ± 0.002 g, HFD 0.070 ± 0.002 g, *p* = .039). (G) HFD male offspring had a lower blood glucose than CON, however this difference was not observed among female offspring (Male CON 3.36 ± 0.014 mmol/L, HFD 2.40 ± 0.27 mmol/L, *p* = .035. Female CON 3.50 ± 0.18 mmol/L, HFD 2.99 ± 0.39 mmol/L, *p* = .332). All values are mean ± SEM and statistical analyses were performed with repeated measures two-way ANOVA, followed by post hoc analysis with Šidák test where applicable (*p < .05, **p < .01, ***p < .001, ****p < .0001).

Dams and offspring from the second mating were included in the final analysis once dam breeding ability was proven. Dams in the HFD group (*n* = 7) gained more weight than CON dams (*n* = 7) and were heavier at mating, during pregnancy and at postnatal days (PN) 1 and 10 (Figure 2A). HFD dams also had higher gestational weight gain (GWG) between mating and E18 than CON dams (HFD vs. CON ± SEM (g) 15.46 ± 1.28 vs. 10.96 ± 1.60, *p* = .048). HFD dams had higher blood glucose levels than CON dams on gestational days 10 and 18 (*n* = 7/group; Figure 2B). Following removal of outliers (based on mean ± 2SD), HFD dams had higher plasma insulin levels when compared with CON dams (Figure 2C) (mean ± SEM (μg/L); HFD, 473.2.8 ± 79.6 (*n* = 4); CON, 128.2 ± 39.6 (*n* = 5); unpaired *t*-test *p* = .004). HFD dams had a longer gestational period (mean ± SEM (days); HFD vs. CON 19.6 ± 0.2 vs. 19.0 ± 0.0; *p* = .04). There were no differences in litter size (HFD vs. CON pups/litter ± SEM; 8.1 ± 0.6 vs. 7.3 ± 0.6; *p* = .33) and no differences in female:male off-spring ratio (HFD vs. CON female:male offspring ratio ± SEM; 0.52 ± 0.16 vs. 0.57 ± 0.15; *p* = .53). Both male and female offspring of HFD dams were lighter than those from CON dams (Figure 2D). There were no differences in brain weights between HFD versus CON off-spring at PN1 (Figure 2E). However, there was a difference in brain:body weight ratio between female HFD and CON offspring suggesting a “brain-sparing” effect in females (Figure 2F). Male HFD off-spring had lower blood glucose compared with CON males, but there were no differences in females (Figure 2G). There were no differences in plasma insulin concentrations between groups in either male or female offspring (HFD vs. CON mean ± SEM (μg/L); male, 154.1 ± 42.3 vs. 140.2 ± 99.8, *p* = .90; female, 156.8 ± 66.2 vs. 69.01 ± 30.8, *p* = .44).

### 3.2 Transcriptional analysis of offspring cortex and cerebellum identified perturbations in genes associated with metabolism and cell differentiation and function

**Figure 3:**
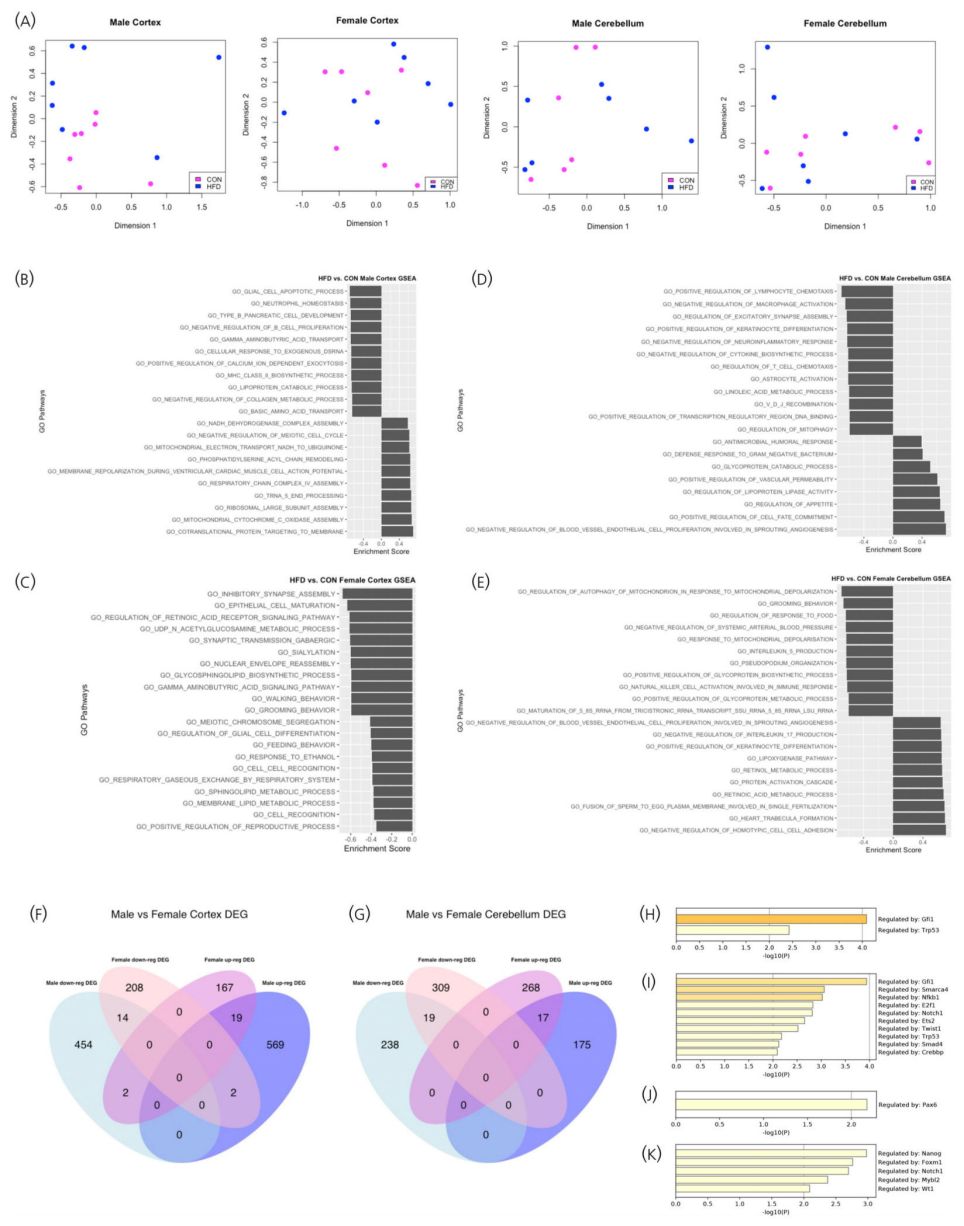
Transcriptomic analysis of offspring cortex and cerebellum. (A) PCA plots of HFD versus CON male and female cortex and cerebellum. There was no clustering between biological replicates within each gender and diet group (purple dots, CON; blue dots, HFD). (B–E) Gene Set Enrichment Analysis of HFD versus CON male (B) and female (C) cortex, and male (D) and female (E) cerebellum. The top 10 upregulated and downregulated pathways have been displayed, except in the female cortex (C) where there were no upregulated pathways. (F, G) Venn diagram demonstrating genes with upregulated (up-reg DEG) and downregulated (down-reg DEG) log2 fold change in HFD versus CON male and female cortex (F) and cerebellum (G) (included genes with *p*-value <.05). (H–K) Transcription factor (TF) analysis demonstrating transcription factors with increased log2fold change in male (H) and female (I) cerebellum. TFs with increased (J) and decreased (K) log2 fold change in male cortex.

Transcriptomic analysis using 3′ RNA-sequencing did not identify any clustering between biological replicates within each diet group (Figure 3A). Additionally, none of the differentially expressed genes (DEGs) between HFD versus CON male and female offspring achieved the threshold for statistical significance (FDR <0.05) in the cortex or cerebellum.

Further analysis to identify overlaps between DEGs in male and female offspring (using *p*-value <.05) demonstrated that the majority of DEGs in males and females were sex-specific in both cortex and cerebellum (Figure 3F,G). Only a small proportion of upregulated genes in HFD male cortex and cerebellum were also upregulated in HFD females, and vice versa. Additionally, several genes which were upregulated in HFD male cortex were downregulated in females, and vice versa. This suggests that the expression of these genes and their vulnerability to the effects of maternal obesity are influenced by offspring sex.

Functional enrichment analysis was performed using the GSEA tool, which compares all DEGs against an a priori defined set of genes known to share similar biological pathways and functions.^42^ Comparison between HFD versus CON male offspring cortex identified enrichment of genes associated with mitochondrial metabolic pathways and gamma aminobutyric acid (GABA) signaling pathways (Figure 3B). Enrichment analysis for the female cortex also revealed alterations in GABA signaling pathways, in addition to downregulation of genes associated with synaptic development and function and metabolic processes, including fatty acid metabolism (Figure 3C). In the cerebellum, there were some similarities between male and female offspring in the pathways altered by exposure to maternal high fat diet, including lipoprotein metabolism. However, there was also evidence of changes in the expression of genes associated with pathways involved in the regulation of excitatory synapse assembly and astrocyte activation in male, but not female, cerebellum (Figure 3D,E).

Finally, transcription factor (TF) analysis using the Transcriptional Regulatory Relationships Unraveled by Sentence-based Text mining database (TRRUST)^44,45^ demonstrated effects of maternal HFD on transcription factors involved in neuronal development in male and female offspring cerebellum (Figure 3H,I). Two TFs were upregulated in male and female cerebellum—growth factor independent 1 gene (*Gfi1*) which is overexpressed in medulloblastoma^46^ and tumor repressor protein 53 (*Trp53*), the expression of which increases following cytotoxic insults and cellular stress.^47^ In the male cortex, the expression of TFs associated with neurogenesis and neuronal maturation, including *Pax6, Nanog*, and *Notch1*, were altered in the HFD group (Figure 3J,K). There were no changes in TF expression in the female HFD cortex.

### 3.3 Exposure to maternal obesity impacts on mRNA expression of DNA methyltransferase and Ten-Eleven Translocase enzymes in male offspring but does not affect global cytosine methylation or hydroxymethylation

**Figure 4:**
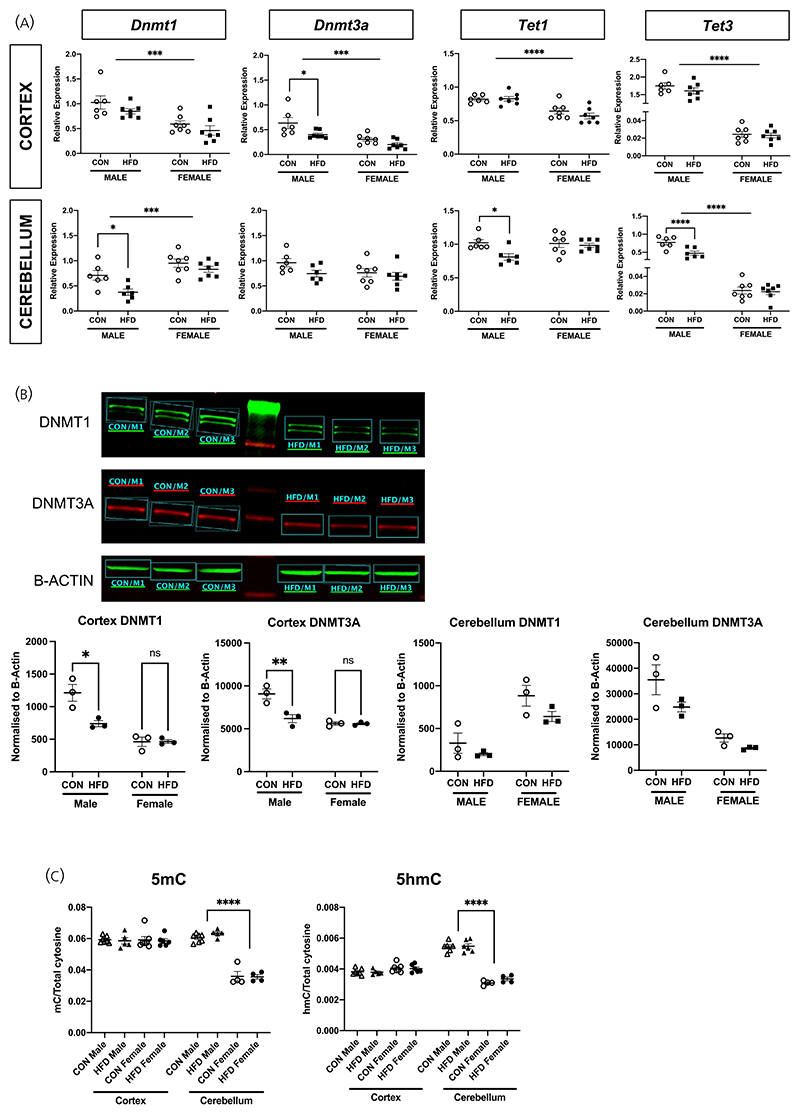
Offspring DNMT and TET enzyme, and 5mC/5hmC levels. (A) RT-qPCR of Dnmt1, Dnmt3a, Tet1, and Tet3 in HFD versus CON male and female cortex and cerebellum. In the cortex, gene expression of Dnmt1 (*F*(1,23) = 21.05, *p* = .0001), Dnmt3a (*F*(1,23) = 20.81, *p* = .0001), Tet1 (*F*(1,23) = 31.44, *p* < .0001), and Tet3 (*F*(1,23) = 744.7, *p* < .0001) in female offspring was lower than males (indicated by horizontal bar). Exposure to HFD did not impact on the expression of these genes in offspring cortex (Dnmt1, *F*(1,23) = 2.947, *p* = .99; Tet1, *F*(1,23) = 0.754, *p* = .394; Tet2, *F*(1,23) = 1.398, *p* = .249), except in males where Dnmt3a expression was lower in HFD vs. CON offspring (*p* = .022, indicated by bracket). In the cerebellum, Dnmt1 expression was higher in female offspring when compared with males (*F*(1,22) = 20.98, *p* = .0001), whereas Tet3 expression was lower in females than in males (*F*(1,22) = 221.9, *p* < .0001) (indicated by horizontal bars). Exposure to maternal HFD associated with reduced expression of Dnmt1 (*p* = .038), Tet1 (*p* = .038), and Tet3a (*p* = .0003) in male (indicated by bracket), but not female, offspring cerebellum (*n* = 6/diet group in males, *n* = 7/diet group in females). (B) Protein expression of both DNMT1 (*p* = .026) and DNMT3A (*p* = .019) was reduced in HFD male cortex when compared with controls (*n* = 3/diet group). There were no differences in DNMT1 and DNMT3A protein expression in HFD versus CON female cortex, as well as cerebellum in both males and females. (C) Global 5mC and 5hmC levels. There were no differences in 5mC and 5hmC levels between diet groups (*F*(1,37) = 0.057, *p* = .813). However, in the cerebellum, 5mC and 5hmC concentration was greater in male vs. female offspring (5mC, *F*(1,37) = 118.5, *p* < .0001; 5hmC, *F*(1,37) = 150.2, *p* < .0001. Indicated by brackets). Unless otherwise stated, repeated measures ANOVA testing was performed.

There were sex differences in the mRNA expression of the DNA methyltransferases (DNMTs), with lower cortical *Dnmt1* and *Dnmt3A* but higher cerebellar *Dnmt1* expression in female offspring compared with males. In males, maternal HFD was associated with a reduction in the mRNA expression of *Dnmt3A* in the cortex and *Dnmt1* in the cerebellum (Figure 4A). There were no differences in females. HFD exposure was associated with a reduction in DNMT1 and 3A protein levels in the male cortex, but there were no differences in the cerebellum (Figure 4B).

There were also sex differences in the mRNA expression of the TET enzymes, with lower expression of *Tet1* in the cerebral cortex and *Tet3* in the cortex and cerebellum in females compared with males (Figure 4A). HFD exposure was associated with a reduction in cerebellar *Tet1* and *Tet3* expression in males but not in females (Figure 4A). There were no differences in the female cortex or cerebellum.

To elucidate any impact of altered *Dnmt* and *Tet* enzyme gene expression on DNA methylation, mass spectrometry was performed to quantify global 5mC and 5hmC levels (Figure 4C). Although there were marked sex differences in 5mC and 5hmC in the cerebellum, with lower 5mC and 5hmC levels in females, there were no effects of HFD exposure in the cortex and cerebellum in either males or females.

## 4 Discussion

In human pregnancies complicated by obesity, maternal blood glucose concentrations increase as the pregnancy progresses^48^ and hyperglycemia and gestational diabetes mellitus (GDM) are more likely to occur from the mid-trimester onwards in obese pregnant women than in women of normal.^49^ These features are recapitulated in this mouse model. The hyperglycemia in obese mouse dams resolved on postnatal day 1, a feature which is similar to that observed in human pregnancies in which the majority of women with GDM return to a normoglycemic state postnatally.^50^

Data from animal models of maternal obesity report both large and small offspring,^51–55^ and in this study, the offspring of high-fat diet dams were lighter than controls. In humans, maternal obesity generally associates with increased offspring birthweight,^56^ with a proportional increase in the risk of delivering large-for-gestational age (LGA) infants with increasing maternal BMI.^57^ This risk is independent of the presence of gestational diabetes.^58,59^ However, there is growing evidence that overweight and obese women,^59–62^ as well as women with GDM,^63,64^ have an increased risk of a small for gestational age (SGA) baby at birth. Our data suggests “brain sparing” in female HFD offspring with preserved brain weight despite a reduction in total body weight. This phenomenon, where there is asymmetrical growth with redistribution of oxygen and nutrients to critical organs (i.e., brain), is frequently observed in conditions associated with placental dysfunction or insufficiency.^65,66^ This “brain sparing” phenomenon in growth restriction has been shown to be associated with poorer neurodevelopmental outcomes when compared with infants who have symmetrical growth restriction.^67,68^

Male HFD offspring had lower blood glucose concentrations on PN1. In humans, neonatal hypoglycemia is a well-documented complication of pregnancies in which the mother has pre-existing or gestational diabetes.^69–71^ Notably, even in the absence of a formal diagnosis of diabetes, maternal hyperglycemia in obese pregnant women is associated with an increased risk of neonatal hypoglycemia.^71^ The mechanisms underpinning the neonatal hypoglycemia may include fetal hyperinsulinemia, which also contributes to the LGA phenomenon in pregnancies complicated by diabetes.^59^ Additionally, following delivery, the sudden cessation of maternal glucose supply and reduced neonatal glycogenolysis may also contribute to early postnatal hypoglycemia.^72^ This is important, since the developing brain depends on glucose as a key source of energy, and neonatal hypoglycemia can have detrimental effects on neurodevelopment.^25,73^ The sex difference in the risk of postnatal hypoglycemia appears to recapitulate observations in humans where male infants of mothers with Type 1 or gestational diabetes have a higher risk of neonatal hypoglycemia.^74,75^ The mechanism(s) underlying the apparent increased male susceptibility to neonatal hypoglycemia may reflect fundamental sex differences in insulin sensitivity.^76^ Consistent with an effect of maternal obesity and hyperglycemia on the availability of substrates including glucose, lactate, and ketone bodies,^53^ gene ontology analysis of DEGs demonstrated an impact on metabolic processes in both cortex and cerebellum.

Perturbations in regulatory processes, including transcription factor and cell signaling activity, may interfere with fundamental cortical and cerebellar developmental milestones and associate with an increased risk of developing neurodevelopmental disorders in later life, including autism spectrum disorders and attention deficit and hyperactivity disorder.^77,78^ This study has focused on offspring cerebral cortex and cerebellum because there is evidence that both structures, and indeed the cortical–cerebellar connectome, are critical in early cognitive development and have been implicated in neurodevelopmental disorders including ASD and ADHD.^26,29,30,77,78^ Gene set enrichment analysis of the transcriptomic data from the cerebral cortex showed alterations in pathways associated with the GABA signaling pathway, synaptic function, and glial cell regulation. Additionally, enrichment analysis of gene expression in male cerebellum identified changes in genes associated with excitatory synapse assembly and astrocyte activation following exposure to a maternal HFD. This is relevant since these pathways are linked to the development of autism spectrum disorders.^27,28,77–79^ Transcription factor analysis demonstrated dysregulation of TFs involved in neurodevelopment in the cerebellum,^80^ some of which associate with psychiatric disorders including schizophrenia. This included the tumor suppressor gene, *Trp53*, which may be important for neuronal protection against neurotoxicity.^81^ Further work is required to establish the implications of these transcriptional changes on the higher prevalence of neurodevelopmental disorders seen in the offspring of obese and diabetic pregnant women.^18,24^

Exposure to a maternal HFD was associated with a reduction in cortical *Dnmt3A* mRNA expression in male offspring and a reduction in both DNMT1 and DNMT3A protein levels in HFD male cortex. In mice, maternal undernutrition resulting in SGA offspring has been associated with reduced DNMT1 protein expression in the hypothalamus and with reduced cell proliferation—particularly in neuronal cells.^82^ In Sprague–Dawley rats, exposure to a maternal high-fat diet was associated with attenuated protein expression of DNMT1 in the newborn hypothalamus.^83^ During development, the TET enzymes also play an important role in the temporal, regional, and functional regulation of neuronal maturation^31^ and there are dramatic changes in gene expression in the perinatal mouse brain, where *Tet* enzyme expression is high during the newborn period and dramatically decreases, at least in the hippocampus and hypothalamus, by postnatal day 7.^84^ We observe a difference in the mRNA expression of *Tet1* and *Tet3* in the cerebellum in males exposed to maternal obesity compared with controls. Although metabolic perturbations secondary to obesity and diabetes impact TET enzyme expression in other organ systems including the placenta,^85–87^ any effects in the developing brain are not well characterized. Although the DNMT and TET enzymes may facilitate the dynamic changes in DNA methylation/hydroxymethylation during neurodevelopment^37^ there were no differences in global 5mC and 5hmC levels between HFD and CON offspring. However, this does not exclude changes in 5mC/5hmC at a single base pair level or alterations in DNA methylation patterns across the different regions in the genome. Further, 5mC/5hmC patterns differ between cell types and between different brain regions^33^ so that analysis of 5mC/5hmC in whole cortex may miss more subtle changes in specific brain subregions.

There were marked sex differences in the response to prenatal exposure to maternal obesity. This is not surprising since known sex differences in the risk of disease in adulthood may be apparent in childhood^88^ and may be influenced by the environment in early life. For example, females with low birthweight may be more vulnerable to an increased risk of cardiovascular disease than males.^89,90^ In contrast, males may be more susceptible to the early life “programming” of neurodevelopmental disorders.^91^ The mechanism(s) underlying sex differences in the vulnerability of different organ systems to the effects of early life programming remain poorly understood. Sex hormones may play a role, and animal studies have provided evidence that the macro- and microscopic differences between male and female brains^92^ may be influenced by androgen exposure, particularly testosterone, in the fetal and neonatal periods in human and non-human primates.^92,93^ Studies have reported sex differences in brain mitochondrial function including NADH-linked respiration, pyruvate dehydrogenase activity and formation of reactive oxygen species^94,95^ which may be mediated by sex steroids, and which associate with lower oxidative stress, at least in adult females.^94^ Thus, it is possible that prenatal sex differences in sex steroid concentrations and metabolism may explain the enrichment in mitochondrial metabolism pathways seen in male, but not female offspring following exposure to maternal HFD in utero.

We also found sex differences in DNMT and TET gene expression in the cortex and cerebellum, and in 5mC and 5hmC levels. Previous studies have shown sex differences in the expression of the TET and DNMT enzymes in several regions of the brain during the neonatal period, including the preoptic area and the hippocampus.^84,96^ DNA methyltransferase activity in the preoptic area is influenced by sex steroids. In female mice, exposure to exogenous estradiol in the early postnatal period leads to the masculinization of CpG methylation patterns in the brain.^96^ Males and “masculinized” females had reduced levels of global DNA methylation when compared with females, in association with altered DNMT activity and impacts on dendritic spine density.^96^ In contrast to the sex differences we found in 5mC levels in the frontal cortex and cerebellum, 5mC levels are higher in the preoptic area of the hypothalamus in females on postnatal day 7,^84^ suggesting that sex differences in 5mC and 5hmC levels differ between brain regions and between developmental periods.

There are several limitations to this study, including the phenotype of the offspring from HFD dams. In humans, obese and diabetic pregnancies commonly associate with offspring macrosomia, and it would be useful to ascertain whether similar transcriptomic changes are observed in LGA offspring. We acknowledge that the small sample sizes in several experiments are described. Nevertheless, we ensured high-quality samples were processed and offspring from independent litters were used as replicates to ensure the reliability of the results.

The lack of clustering between biological replicates may be due to the cellular heterogeneity in the developing brain. The transcriptomic data was obtained from bulk RNA-sequencing, and cell sorting before sequencing may provide us with a better resolution of cell-specific transcriptomic changes. The neonatal hypoglycemia observed in off-spring of HFD dams may in itself impact offspring neurodevelopment. It would, therefore, be useful to dissect the impact of hypoglycemia versus hyperglycemia using cell culture models and manipulation of glucose availability to assess the impact of (1) hypoglycemia on astrocytes and neurons, and (2) hypoglycemia on astrocytes and neurons that were subjected to prior exposure to hyperglycemic conditions. Behavioral studies to establish the impact of these transcriptional changes on offspring neurodevelopment will allow us to better understand the mechanism(s) underlying the neurodevelopmental and psychiatric disorders seen in the offspring of obese and diabetic pregnant women. Further, it will expand on our current knowledge of the sexual dimorphism seen in these disorders.

To conclude, this study utilized a murine diet-induced obesity model to demonstrate that the exposure of the offspring to maternal high fat diet during the perinatal period associates with offspring growth restriction with brain sparing and male offspring postnatal hypoglycemia which, to an extent, recapitulates that observed in human pregnancies.^60,69,70^ This study has also highlighted the sexual dimorphism in the transcriptional landscape in offspring cortex and cerebellum following exposure to maternal obesity and hyperglycemia. The altered mitochondrial metabolic pathways seen in male, but not female, cortex suggest that there may be a male susceptibility to altered metabolic milieu during the in utero and early postnatal period. This study also demonstrated a sex difference in the expression of key enzymes involved in DNA methylation in the early postnatal period, including DNMT and TET enzymes. Further work to explore the mechanism(s) underpinning these differences may allow us to understand some of the gender disparities associated with risks of neurodevelopmental and psychiatric disorders.

## Supplementary Material

Supporting Information

## Figures and Tables

**Table 1:** List of primers for qPCR.

Gene	Sequence	Probe
TBP	For: 5′-GGCGGTTTGGCTAGGTTT-3′ Rev: 5′-TCTGGGTTATCTTCACACACCA-3′	107
DNMT1	For: 5′-GCTACCAGTGCACCTTTGGT-3′ Rev: 5′-ATGATGGCCCTCCTTCGT-3′	1
TET1	For: 5′-GGCTCCAGTTGCTTATCAAAA-3′ Rev: 5′-CCCTCTTCATTTCCAAGTCG-3′	67
TET3	For: 5′-AAGACGCCACGAAAGTTCC-3′ Rev: 5′-TGAAAGCTATTCCGGAGCAC-3′	63
DNMT3a	For: 5′-TACCAGTATGACGACGATGG-3′ Rev: 5′-GGGCATAAGGGCACCTAT-3′	-
RPL30	For: 5′-CTGCTCTCAAGGTTGTTCG-3′ Rev: 5′-GCCCTCAAGGTTGTGC-3′	-

## Data Availability

The data that support the findings of this study are available from the corresponding author upon reasonable request.
