# Supplementary material for: Maternal high fat and high sugar diet impacts on key DNA methylation enzymes in offspring brain in a sex‐specific manner
Source: J Neuroendocrinol. 2025 May 15;37(8):e70046. doi: 10.1111/jne.70046 (PMC7617894; doi:10.1111/jne.70046)
Supplement: Supplementary file 1 — Data S1. Supporting Information. [file JNE-37-e70046-s001.docx]

**Supplement 1**

List of R packages

| edgeR | Version 3.28.1 |
| --- | --- |
| tidyverse | Version 1.3.0 |
| ggplot2 | Version 3.3.3 |
| VennDiagram | Version 1.6.20 |
